# Profiling Y561-Dependent and -Independent Substrates of CSF-1R in Epithelial Cells

**DOI:** 10.1371/journal.pone.0013587

**Published:** 2010-10-26

**Authors:** Melodie L. Knowlton, Laura M. Selfors, Carolyn N. Wrobel, Ting-Lei Gu, Bryan A. Ballif, Steven P. Gygi, Roberto Polakiewicz, Joan S. Brugge

**Affiliations:** 1 Department of Cell Biology, Harvard Medical School, Boston, Massachusetts, United States of America; 2 Cell Signaling Technology, Inc., Danvers, Massachusetts, United States of America; Roswell Park Cancer Institute, United States of America

## Abstract

Receptor tyrosine kinases (RTKs) activate multiple downstream cytosolic tyrosine kinases following ligand stimulation. SRC family kinases (SFKs), which are recruited to activated RTKs through SH2 domain interactions with RTK autophosphorylation sites, are targets of many subfamilies of RTKs. To date, there has not been a systematic analysis of the downstream substrates of such receptor-activated SFKs. Here, we conducted quantitative mass spectrometry utilizing stable isotope labeling (SILAC) analysis to profile candidate SRC-substrates induced by the CSF-1R tyrosine kinase by comparing the phosphotyrosine-containing peptides from cells expressing either CSF-1R or a mutant form of this RTK that is unable to bind to SFKs. This analysis identified previously uncharacterized changes in tyrosine phosphorylation induced by CSF-1R in mammary epithelial cells as well as a set of candidate substrates dependent on SRC recruitment to CSF-1R. Many of these candidates may be direct SRC targets as the amino acids flanking the phosphorylation sites in these proteins are similar to known SRC kinase phosphorylation motifs. The putative SRC-dependent proteins include known SRC substrates as well as previously unrecognized SRC targets. The collection of substrates includes proteins involved in multiple cellular processes including cell-cell adhesion, endocytosis, and signal transduction. Analyses of phosphoproteomic data from breast and lung cancer patient samples identified a subset of the SRC-dependent phosphorylation sites as being strongly correlated with SRC activation, which represent candidate markers of SRC activation downstream of receptor tyrosine kinases in human tumors. In summary, our data reveal quantitative site-specific changes in tyrosine phosphorylation induced by CSF-1R activation in epithelial cells and identify many candidate SRC-dependent substrates phosphorylated downstream of an RTK.

## Introduction

Growth factors and their cognate receptor tyrosine kinases (RTK) are key regulators of tumor cell initiation and progression [1]. Growth factor binding and subsequent RTK auto-phosphorylation lead to the activation of pathways that regulate cell proliferation, survival, growth, adhesion and motility. Inappropriate RTK activation can drive tumor cell growth, survival, invasion and metastasis. RTKs like epidermal growth factor receptor (EGFR) and epidermal growth factor receptor 2 (Her2/ERBB2) are overexpressed or activated in a variety of human cancers [2]. In non-small cell lung cancers (NSCLC), activating *EGFR* mutations are found in 10–15% of Caucasian and 30–40% Asian patients [3], [4]. ERBB2, as a further example, is upregulated by gene amplification in 15–30% of invasive mammary ductal cancers [1]. RTKs are known to activate several downstream tyrosine kinases, including members of the SRC, ABL, and JAK kinase families. These cytosolic kinases make a significant contribution to the dramatic increase in tyrosine phosphorylation induced by RTKs. It has been difficult to define the precise subset of proteins targeted by any individual tyrosine kinase that is a component of these kinase signaling cascades due to the overlap in substrates phosphorylated by activated RTKs and non-receptor tyrosine kinases. Previous studies have utilized activated mutant variants of non-receptor tyrosine kinases like SRC to identify downstream substrates of this kinase subfamily; however, these overexpressed, constitutively active mutants likely display promiscuous activities that do not necessarily reflect the substrates of the endogenous protein when activated by an upstream RTK.

SRC and other SRC family kinases (SFKs) are activated downstream of many different RTKs [5]. SRC activity is critical for several phenotypic events induced by RTK activation including DNA synthesis, cytoskeletal reorganization and disruption of cell-cell adhesion [6], [7], [8]. In human tumors, RTK activation of SFKs may contribute to tumor progression and lead to more aggressive tumor phenotypes [9]. Dominant negative SRC mutants, pharmacological inhibition of SRC kinase activity, and SRC-specific docking site RTK mutants have been used to address the specific role of SRC in RTK signal transduction [10]. Studies in breast cancer models using these methods have demonstrated that inhibition of SRC kinase activity suppresses phenotypic effects induced by the overexpression or activation of RTKs like EGFR and ERBB2, e.g. anchorage-independent growth, motility and survival [10], [11], [12], [13]. Therefore, RTK-induced SRC activity drives aspects of RTK signaling important in tumor progression, and the identification of RTK-induced SRC substrates will offer further insight into the role of SRC in tumorigenesis.

We previously demonstrated [8] that Src activation regulates a subset of phenotypic alterations induced by the colony stimulating factor 1 receptor tyrosine kinase (CSF-1R), which has been implicated in the progression of multiple types of carcinoma including breast cancer [14], [15], [16], [17], [18], [19], [20]. SRC activity was found to be critical for CSF-1R-induced disruption of cell-cell adhesion of MCF-10A cells, immortalized, non-transformed mammary epithelial cells [8]. These alterations are associated with loss of plasma membrane association of E-cadherin, a key regulator of epithelial intercellular adhesion. The CSF-1R tyrosine phosphorylation site that binds to SRC and other SFKs, Y561, was found to be critical for disruption of cell-cell adhesion [8], [21]. Expression of a dominant-negative mutant of SRC (c-SRC K295R) or pharmacological inhibition of SRC kinase activity in the presence of CSF-1R activation preserves cell-cell adhesion, providing additional evidence that SRC activity and phosphorylation of SRC substrates are involved in the disruption observed [8].

Few studies have examined the specific set of SRC substrates phosphorylated after RTK activation [10], [22]. A recent mass spectrometry (MS) analysis by Amanchy and colleagues identified 43 candidate SRC substrates downstream of platelet-derived growth factor (PDGF) receptor in fibroblasts using SU6656, a pharmacological inhibitor of SRC kinase activity [22]. While this work identified candidate substrates of endogenous SRC, all cellular SRC family kinases were inhibited by this inhibitor, so this analysis did not distinguish substrates that are directly downstream of PDGFR activation from those regulated by adhesion receptors and other cellular processes. Several MS analyses have identified proteins that display increased tyrosine phosphorylation in cells overexpressing wild-type SRC or an activated mutant variant [23], [24], [25], [26]. However, the relevance of these targets in human tumors is not clear, as SRC is not frequently mutated or overexpressed in breast cancers, and it is more likely activated in breast carcinomas by an upstream activated RTK or another receptor known to couple to SRC [5], [10], [11], [13], [27].

Using quantitative MS analysis, we examined CSF-1R-induced tyrosine phosphorylation using quantitative PhosphoScan® analysis. PhosphoScan® analysis is an MS approach that utilizes immunoaffinity purification of tyrosine phosphorylated peptides prior to MS analysis to enrich their representation in the sample [23]. We profiled site-specific changes in tyrosine phosphorylation induced by activated CSF-1R and activated CSF-1R defective for the recruitment of SFKs in MCF-10A cells. To do this, we utilized a previously described constitutively active variant of CSF-1R (CA-CSF-1R) and CA-CSF-1R with a tyrosine to phenylalanine substitution at Y561 (CA-CSF-1R Y561F), the recruitment site for SRC and SFKs [8]. This phosphotyrosine proteomic MS analysis of parental MCF-10As and cells expressing the two CSF-1R variants allowed us to characterize CSF-1R-induced tyrosine phosphorylation events and to distinguish Y561-independent and Y561-dependent phosphorylation events stimulated by the activated CSF-1R. The Y561-dependent substrates revealed a set of putative SRC substrates and subsequent bioinformatics analyses of these targets in human tumor datasets identified potential biomarkers of SRC activation in human tumors.

## Results

### Quantitative MS analysis

**Figure 1:**
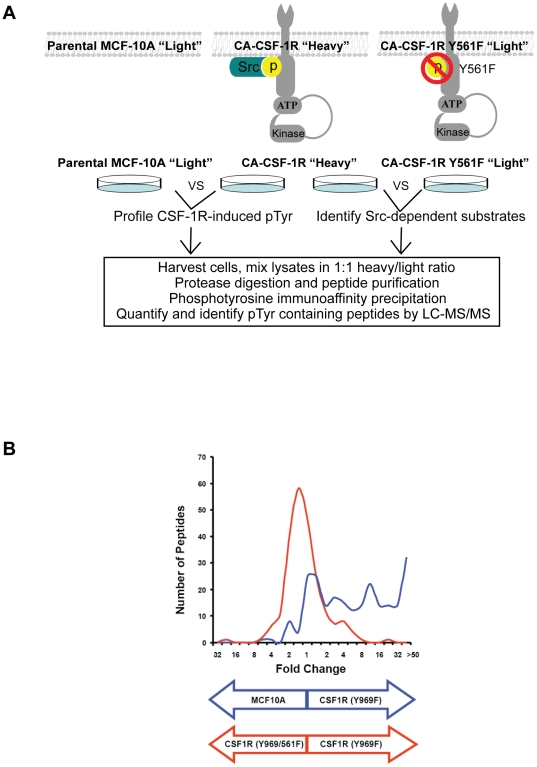
Quantitative MS profiles CSF-1R-induced and SRC-dependent changes in tyrosine phosphorylation in MCF-10A cells. **A**) Schematic of experimental design showing the CSF-1R constitutively active mutants used in MCF-10A cells to profile changes in tyrosine phosphorylation and the protocol followed for quantitative MS analysis **B**) Graph of data from quantitative MS analysis depicting the distribution of peptides relative to the fold change in tyrosine phosphorylation levels. Blue: CA-CSF-1R vs. parental MCF-10A; Red: CA-CSF-1R vs. CA-CSF-1R Y561F MCF-10A.

To systematically profile changes in tyrosine phosphorylation downstream of CSF-1R activation and identify candidate SRC substrates, we conducted quantitative liquid chromatography and tandem MS (LC-MS/MS) analysis using metabolic labeling with stable isotopes (SILAC) together with phosphotyrosine proteome analysis using samples derived from MCF-10A cells expressing either CA-CSF-1R or CA-CSF-1R Y561F [23]. For quantitative analysis using SILAC, one experimental sample is labeled with “heavy” amino acids, while the other sample is grown in the presence of “light” amino acids. In this study, CA-CSF-1R-expressing MCF-10A cells were labeled with “heavy” ^13^C_6_-^15^N_2_-L-lysine and ^13^C_6_-^15^N_4_-L-arginine. Cells expressing CA-CSF-1R Y561F and parental MCF-10A cells were labeled with the corresponding “light” isotopic variants of the same amino acids (Figure 1A). Naturally occurring amino acid isotopic variants were eliminated from the serum supplementing the growth medium by dialysis, and cells were passaged a minimum of five times in the labeling medium to ensure complete incorporation of the labeled amino acid. Before harvesting for phosphotyrosine proteome analysis, the cells were cultured in the absence of EGF, as EGF-starvation leads to dominant CSF-1R signaling in MCF-10A cells overexpressing the CSF-1R [8]. Samples were mixed in a 1∶1 “heavy/light” ratio, proteolytically digested, and subjected to phosphotyrosine immunoprecipitation prior to LC-MS/MS.

We quantitatively assessed the tyrosine phosphorylation of 275 peptides in the comparison of CA-CSF-1R MCF-10A cells to parental MCF-10A cells (Table S1), and 324 peptides in the comparison of CA-CSF-1R MCF-10A cells to CA-CSF-1R Y561F MCF-10A cells (Table S2). The fold differences in tyrosine phosphorylated peptides were calculated by determining the ratio of tyrosine phosphorylated peptides detected in each of the cell pairs e.g., CA-CSF-1R vs. parental MCF-10A cells or CA-CSF-1R vs. CA-CSF-1R Y561F cells (Figure 1A). Figure 1B illustrates the distribution of the identified phosphopeptides for each comparison (CA-CSF-1R/MCF-10A; CA-CSF-1R/CA-CSF-1R Y561F) relative to the fold change differences in tyrosine phosphorylation.

### CSF-1R induced changes in phosphorylation

To guide our assignment of the threshold for significant fold changes phosphorylation, we first graphed the distribution of the non-phosphorylated peptides present in the immunoprecipitated samples and determined the mean of the quantitative fold change values. We assumed data normality based on these distributions; therefore, fold change values greater than two deviations from the mean are considered 95% likely to represent significant differences in tyrosine phosphorylation. Each comparison had a different mean value for the non-phosphopeptide distribution; however, the same statistical threshold was applied to both comparisons. For the CA-CSF-1R/parental MCF-10A comparison, fold change ratios >1.94 were defined as an upregulation in the levels of tyrosine phosphorylation, ratios 0.64–1.94 as unchanged, and ratios <0.64 as a downregulation of tyrosine phosphorylation.

100 tyrosine sites on 74 proteins were phosphorylated above threshold level in cells expressing CA-CSF-1R relative to parental MCF-10A cells and were defined as “CSF-1R-induced” (Table 1). The threshold value (ratio >1.94) used in this analysis was based on a statistical analysis comparing non-phosphorylated and phosphorylated peptides detected in the screen (see Materials and Methods). Five tyrosine sites on four proteins exhibited lower tyrosine phosphorylation in CA-CSF-1R cells and were classified as “CSF-1R downregulated”. Forty-five tyrosine residues on 36 proteins exhibited no change in the level of tyrosine phosphorylation upon CSF-1R activation and were labeled as “basally phosphorylated” and are likely substrates of kinases basally active in MCF-10A cells (Table S3).

**Table 1:** Peptides with CSF-1R induced tyrosine phosphorylation in MCF-10A cells.

Protein Name	UniProt#	NCBI Site	Fold change* (CA-CSF-1R/MCF-10A)	Fold change* (CA-CSF-1R/Y561F)	Src regulation
Transferrin receptor	P02786	Y20	313.67	-	
APLP2	Q06481	Y750	73.37	-	
D-Prohibitin	Q99623	Y248	>55.42	-	
PAR3	Q8TEW0	Y489	>34.42	-	
PKM2	P14618-2	Y105	29.01	-	
PIK3R1	P27986	Y470	>24.59	-	
PIK3R1	P27986	Y607	>22.45	-	
PAR3	Q8TEW0	Y719	19.88	-	
LAPTM4A	Q15012	Y230	>18.02	-	
TAOK1	Q7L7X3	Y309	>17.57	-	
PIK3R2	O00459	Y577	>13.95	-	
Beta-adaptin	P63010	Y277	>12.57	-	
UBXD8	Q96CS3	Y79	>12.42	-	
UQCRC2	P22695	Y207	>10.13	-	
HSP90-beta	P08238	Y483	9.54	-	
CCT-theta	P50990	Y29	9.4	-	
Syndecan-4	P31431	Y197	6.8	-	
CSF-1R	P07333	Y556	>5.68	-	
CDC2	P06493	T14	4.18	-	
EphA2	P29317	T593	3.58;3.58	-	
CDC2	P06493	Y19	3.36	-	
IRS2	Q9Y4H2	Y598	3.27	-	
CASKIN-2	Q8WXE0	Y253	3.21	-	
Desmoglein	Q14126	Y1013	2.06	-	
EphB4	P54760	Y774	2.03;2.03;2.03	1.75;1.75	Src-dependent
FLJ20625	Q9NWT0	Y40	>19.60	2.06	Src-dependent
EphA2	P29317	Y575	3.28	2.11	Src-dependent
Beta-adaptin	P63010	Y276	>11.05;26.66	2.12	Src-dependent
KIAA0323	O15037	Y456	>14.07;150.39;105.93	2.22;1.98;1.98;1.7	Src-dependent
Intersectin 2	Q9NZM3	Y552	51.99	2.73;2.73	Src-dependent
ACK	Q07912	Y518	2.29	3.45	Src-dependent
Hrs	O14964	Y216	148.61	3.51;3.5	Src-dependent
ZDHHC7	Q9NXF8	Y130	>50.78;173.17	4.05;3.99;3.98	Src-dependent
TJAP1	Q5JTD0	Y352	>88.49	4.31	Src-dependent
Hrs	O14964	Y334	>34.54;229.48	4.49	Src-dependent
Hrs	O14964	Y132	>296.57;1823.09;28.34;8.78	5.06;3.12;3.12;3.11;3.11;2.63;2.63	Src-dependent
p120 catenin	O60716	Y904	3.27;3.27	5.31;5.31	Src-dependent
CSF-1R	P07333	Y561	>37.45	>16.44	Src-dependent
STAT3	P40763-2	Y704	4.85;4.45;4.45	0.73;0.72;0.72	Src-independent
Plakophilin 3	Q9Y446	Y176	2.37;2.37	0.74	Src-independent
RPS10	P46783	Y12	10.35	0.74;0.74	Src-independent
STAT3	P40763	Y705	4.76;4.76;4.64;4.64	0.77;0.77	Src-independent
Insulin Receptor	P06213	Y1189	2.41	0.84;0.84;0.73	Src-independent
Talin 1	Q9Y490	Y70	31.66	0.84	Src-independent
ACLY	P53396	Y1073	>26.05;>11.93	0.9;0.9	Src-independent
SHB	Q15464	Y246	2.78	0.93	Src-independent
LDLR	P01130	Y845	61.43;32.64	1;1	Src-independent
CSF-1R	P07333	Y809	>45.71;>20.07;1292.56;10.6	1.01;1.01	Src-independent
EphA2	P29317	Y588	4.61	1.07	Src-independent
SHB	Q15464	Y268	2.91;2.91	1.09;1.09	Src-independent
Calmodulin	P62158	Y99	127.33;70.6	1.11;1.11;0.93;0.93	Src-independent
PTTG1IP	P53801	Y174	>30.11;17.83	1.15;1.11;1.11	Src-independent
EphA2	P29317	Y772	3.79;3.32;3.32	1.16;1.16;1.09;1.09	Src-independent
Integrin beta-4	P16144	Y1207	2.33;2.33;1.39;1.39	1.18	Src-independent
GSTP1	P09211	Y7	35.59	1.19;1.19	Src-independent
eEF1A-2	Q05639	Y29	3.45	0.65;0.65	Src-independent
PKC, Delta	Q05655	Y374	>9.84	0.65	Src-independent
RPS27	P42677	Y31	7.36	0.66;0.66	Src-independent
CDC2	P06493	T14, Y15	5.41;5.27	0.67;0.58	Src-independent
Vimentin	P08670	Y53	2.15	0.68	Src-independent
PIK3R1	P27986	Y467	6.15	0.68;0.64;0.64	Src-independent
Annexin A2	P07355	Y29	12.46;5.27;5.27	0.69;0.69	Src-independent
KIAA1217	Q5T5P2	Y244	3.08;3.07	0.7;0.7	Src-independent
PIN4	Q9Y237	Y122	>21.65	0.75;0.68;0.68	Src-independent
CSF-1R	P07333	Y699	>8.52;>38.88;>32.34;>13.82	0.76;0.74;0.73;0.68;0.66;0.66;0.66;0.62;0.56;0.56	Src-independent
CSF-1R	P07333	Y923	>133.30	0.8;0.63	Src-independent
Ack	Q07912	Y857	4.37	1.13; 1.01; 1.01	Src-independent
EphA2	P29317	Y588, Y594	4.53;3.66	1.16; 0.62; 0.62	Src-independent
CSF-1R	P07333	Y873	>56.43;>38.15	1.3; 1.3; 1.21	Src-independent
Caveolin 1	Q03135	Y14	3.53;2.92;2.74	0.67;0.57;0.53	Src-inhibited
SLC20A1	Q8WUM9	Y388	>13.04	0.25	Src-inhibited
HGK	O95819	Y1227	>15.71	0.28;0.28	Src-inhibited
GAB1	Q13480	Y659	16.82	0.31	Src-inhibited
ERK2	P28482	T184, Y186	56.77	0.31;0.31	Src-inhibited
PTRF	Q6NZI2	Y308	6.84;6.13;0.96;0.94	0.35;0.35;0.34;0.28;0.28	Src-inhibited
Annexin A1	P04083	Y38	19.83	0.42;0.42	Src-inhibited
H2B2E	Q16778	Y43	>31.63;1152.72	0.43;0.42;0.42	Src-inhibited
Plakophilin 3	Q9Y446	Y84	67.23;57.48	0.43;0.43;0.42;0.42	Src-inhibited
ARP2/3 Subunit 3	O15145	Y46	>9.76	0.44	Src-inhibited
PDLIM5	Q96HC4	Y251	5.23	0.49	Src-inhibited
UrdPase 1	Q16831	Y35	16.85	0.49	Src-inhibited
p120 catenin	O60716	Y96	2.33	0.5	Src-inhibited
SHC1	P29353	Y427	5.87;5.87;5.53;5.53	0.51	Src-inhibited
FAM62A	Q9BSJ8	Y822	7.99	0.51	Src-inhibited
ERK1	P27361	Y204	27.99	0.52	Src-inhibited
Annexin A2	P07355	Y315	236.05	0.52;0.52	Src-inhibited
CDK3	Q00526	Y15	7.46;4.62;4.61	0.53;0.53;0.43;0.21;0.21	Src-inhibited
Annexin A2	P07355	Y237	11.78;11.75;10.71	0.53;0.53;0.47	Src-inhibited
ERK2	P28482	Y186	35.57;18.11	0.53;0.53;0.47	Src-inhibited
Enolase 1	P06733	Y188	2.46	0.54	Src-inhibited
Annexin A2	P07355	Y23	3.3;3.3;2.96	0.56;0.56;0.56;0.56;0.55;0.55	Src-inhibited
SLC38A2	Q9HAV3	Y41	21.5	0.56;0.56	Src-inhibited
PIK3R1	P27986	Y580	>56.64;79.89;36.8;36.73	0.57;0.57;0.57;0.56;0.56; 0.54;0.54;0.52;0.51;0.51	Src-inhibited
DLG1	Q12959	Y760	>11.65	0.59;0.51	Src-inhibited
DLG3	Q92796	Y673	>12.59;128.61	0.59;0.59	Src-inhibited
VASP	P50552	Y38	109.16	0.59	Src-inhibited
LDH-A	P00338	Y238	18.81	0.6;0.6	Src-inhibited
Vimentin	P08670	Y61	2.01	0.61	Src-inhibited
PZR	O95297	Y263	12.21;10.01;7.27;7.27	0.62;0.62;0.6;0.6	Src-inhibited
NHP2L1	P55769	Y31	>16.86	0.62	Src-inhibited
Cdc2	P06493	Y15	4.69;4.69;4.18	0.79;0.61;0.55;0.55;0.55;0.49;0.49	Src-inhibited
H2B1L	Q99880	Y42	>113.37	0.53;0.53;0.42;0.42	Src-inhibited
CNP	P09543	Y110	2.8	0.48; 0.48; 0.44	Src-inhibited
CDK3	Q00526	Y19	870.42;4.62	0.52; 0.52	Src-inhibited

*Multiple peptides were detected for tyrosine sites with multiple values.

An additional 38 tyrosine sites on 36 proteins were found to be phosphorylated in the CA-CSF-1R vs. CA-CSF-1R Y561F analysis, but not detected in the CA-CSF-1R vs. parental MCF-10A analysis. These sites were denoted “CSF-1R induced*” (Table 2). It remains a possibility that these sites are not CSF-1R induced, but were undetectable in the parental MCF-10As from the CA-CSF-1R vs. MCF-10A MS analysis. However, these sites were denoted as CSF-1R-induced* to distinguish them as qualified CSF-1R regulated sites (Table 2). Where these phosphorylated tyrosine residues are included in later comparative analyses they are clearly distinguished. Inclusion of these sites adds 24 CSF-1R induced* proteins (twelve proteins had other tyrosine sites that were unambiguously CSF-1R induced). Further validation will be necessary to determine whether this subset of proteins is regulated by CSF-1R activation. Overall, we identified 98 proteins with either CSF-1R-induced (74 proteins) or CSF-1R induced* (24 proteins) tyrosine phosphorylation.

**Table 2:** Proteins with CSF-1R induced* tyrosine phosphorylation detected in CA-CSF-1R v CA-CSF-1R Y561F analysis.

Protein Name	UniProt#	NCBI Site	Fold change	Src regulation
STAT 5A	P42229	Y694	2.11;2.11	Src-dependent
EphB4	P54760	T587, Y595	2.09	Src-dependent
RAN	P62826	Y146	1.81	Src-dependent
SPG20	Q8N0X7	Y46	1.66	Src-dependent
RPS13	P62277	Y37	1.49	Src-independent
PIK3R3	Q92569	Y373	1.43	Src-independent
HGF-R	P08581	Y1234	1.21;1.21	Src-independent
Cofilin	P23528	Y139	1.14;0.79	Src-independent
p38-alpha	Q16539-2	Y182	1.03;1.03	Src-independent
Insulin Receptor	P06213	Y1185, Y1189	0.77;0.77	Src-independent
EphA2	P29317	Y594	0.74;0.74	Src-independent
Transferrin receptor	P02786	S19	1.28	Src-independent
LDH-B	P07195	Y239	1.23	Src-independent
KIRREL	Q96J84	Y724	1.17	Src-independent
FLJ20625	Q9NWT0	Y140	1.13	Src-independent
GUK1	Q16774	Y52	1.03	Src-independent
Yes	P07947	Y221	0.99	Src-independent
D-Prohibitin	Q99623	Y121	0.95	Src-independent
MGC14839	Q96A22	Y78	0.92	Src-independent
ADK2	P54819	Y200	0.86	Src-independent
ADK2	P54819	Y199	0.86	Src-independent
KIRREL	Q96J84	Y605	0.85	Src-independent
Crk	P46108	Y221	0.77	Src-independent
Caveolin 1	Q03135	Y25	0.69	Src-independent
Pyk2	Q14289	Y579, Y580	0.67	Src-independent
STEAP1	Q9UHE8	Y27	0.66	Src-independent
KRT81	Q14533	Y282	0.64	Src-independent
CDK3	Q00526	T14, Y15	0.61;0.61	Src-inhibited
NDUFB10	O96000	Y54	0.62	Src-inhibited
SHC1	P29353	Y350	0.62	Src-inhibited
PGK1	P00558	Y196	0.61	Src-inhibited
EGF-R	P00533	Y1197	0.6	Src-inhibited
ZDHHC5	Q9C0B5	S529 Y533	0.53	Src-inhibited
PI3K p110 alpha	P42336	Y508	0.45	Src-inhibited
GAB1	Q13480	Y406	0.27	Src-inhibited
MGC32065	Q8WV41	Y264	0.22	Src-inhibited
H2B1L	Q99880	Y40	0.43	Src-inhibited
PTRF	Q6NZI2	Y156	0.45;0.45	Src-inhibited

As expected, CSF-1R activation in parental MCF-10A cells induced a strong increase in tyrosine phosphorylation. Proteins previously shown to be tyrosine phosphorylated following CSF-1R activation were detected in our analysis (Table 3), thus validating this approach for identification of CSF-1R substrates. These included STAT3, PKCδ, vimentin, SHC1, annexin A1, multiple isoforms of PI3K p85-alpha, ERK1, ERK2, and tyrosine residues on the CSF-1R (Y556, Y561, Y699, Y809, Y873, Y923) [28], [29], [30]. Similarly, the CSF-1R-induced* set of proteins included STAT5A, YES, PYK2 and p38-alpha, which are known to be phosphorylated downstream of CSF-1R (Table 1).

**Table 3:** Profile of known CSF-1R induced changes in tyrosine phosphorylation in MCF-10A cells.

Protein Name	UniProt #	NCBI Site	Fold change (CA-CSF-1R/MCF10A)	Function
Annexin A1	P04083	Y38	19.83	Calcium-binding protein
CSF-1R	P07333	Y923	>133.30	Receptor tyrosine kinase (RTK)
CSF-1R	P07333	Y873	>56.43	RTK
CSF-1R	P07333	Y809	>45.71	RTK
CSF-1R	P07333	Y699	>38.88	RTK
CSF-1R	P07333	Y873	>38.15	RTK
CSF-1R	P07333	Y561	>37.45	RTK
CSF-1R	P07333	Y699	>32.34	RTK
CSF-1R	P07333	Y809	>20.07	RTK
CSF-1R	P07333	Y699	>13.82	RTK
CSF-1R	P07333	Y699	>8.52	RTK
CSF-1R	P07333	Y556	>5.68	RTK
CSF-1R	P07333	Y809	1292.56	RTK
ERK 1	P27361	Y204	27.99	Protein kinase, Ser/Thr
ERK 2	P28482	T184, Y186	56.77	Protein kinase, Ser/Thr
PIK3R1	P27986	Y580	>56.64	Kinase, lipid
PIK3R1	P27986	Y470	>24.59	Kinase, lipid
PIK3R1	P27986	Y607	>22.45	Kinase, lipid
PIK3R1	P27986	Y580	79.89	Kinase, lipid
PIK3R1	P27986	Y580	36.8	Kinase, lipid
PIK3R1	P27986	Y580	36.73	Kinase, lipid
PIK3R1	P27986	Y467	6.15	Kinase, lipid
PIK3R2	O00459	Y577	>13.95	Kinase, lipid
PKC delta	Q05655	Y374	>9.84	Protein kinase, Ser/Thr
SHC1	P29353	Y427	5.87	Adaptor/scaffold
SHC1	P29353	Y427	5.87	Adaptor/scaffold
SHC1	P29353	Y427	5.53	Adaptor/scaffold
SHC1	P29353	Y427	5.53	Adaptor/scaffold
STAT3	P40763	Y705	4.76	Transcription factor
STAT3	P40763	Y705	4.76	Transcription factor
STAT3	P40763	Y705	4.64	Transcription factor
STAT3	P40763	Y705	4.64	Transcription factor
Vimentin	P08670	Y61	2.01	Cytoskeletal protein
Vimentin	P08670	Y53	2.15	Cytoskeletal protein

**Figure 2:**
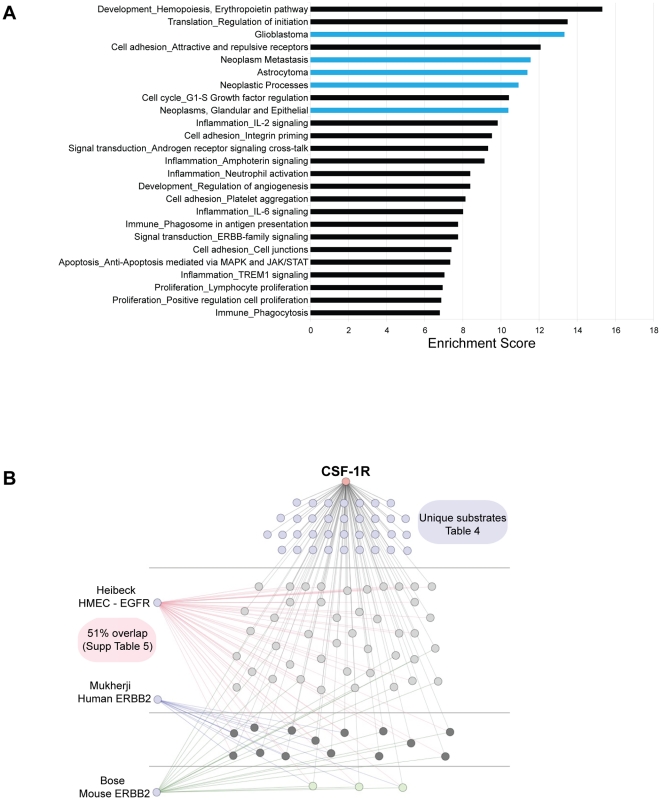
CSF-1R-induced changes in tyrosine phosphorylation. **A**) GeneGO® enrichment analysis of proteins with CSF-1R-induced and CSF-1R-induced* tyrosine phosphorylation. The 20 most significant GeneGO® Processes (black) and 5 most significant diseases (blue) are shown. Enrichment scores are −log(p-value). **B**) Diagram depicting the overlap in phosphorylated proteins between the CSF-1R, EGFR and ERBB2 pathways. Thirty-five proteins were unique to CSF-1R (blue), nineteen were in one other pathway (light gray), fourteen were in two (dark gray), and three were common to all studies (green). The EGFR/CSF-1R overlapping proteins are listed in Table S5.

GeneGO® enrichment analysis of the CSF-1R-induced and CSF-1R-induced* protein set revealed that CSF-1R induces phosphorylation of proteins associated with cellular processes including cell adhesion, developmental pathways, translation initiation, cell cycle regulation, and cytoskeletal dynamics (Figure 2A). Interestingly, CSF-1R activation stimulates phosphorylation of proteins involved in immune and inflammation pathways e.g., interleukin (IL)-6 and IL-2 signaling and neutrophil activation. Phosphorylation of these targets in MCF-10A cells may reflect the conserved function of CSF-1R in the regulation of monocyte cell differentiation and survival. Proteins with CSF-1R-induced tyrosine phosphorylation that contribute to the enriched biological processes are listed in Table S4. We also analyzed the CSF-1R-induced and CSF-1R-induced* proteins for significant association with disease and found a statistically significant enrichment for proteins involved in tumor metastasis and progression (Figure 2A, Table S4).

### Comparative phosphoproteomics – EGFR and ERBB2-induced tyrosine phosphorylation

MCF-10A cells over-expressing CA-CSF-1R are able to proliferate and survive in the absence of EGF, suggesting that CSF-1R and EGFR activate redundant signaling pathways to some degree [8]. The quantitative MS data show that under EGF-deficient experimental conditions phosphorylation of EGFR on Y1172 was equivalent in parental MCF-10As and CA-CSF-1R MCF-10As (Table S1). This data indicates that CSF-1R activation does not influence EGFR directly, but independently activates downstream pathways that promote proliferation and survival.

To identify phosphorylated targets common to CSF-1R and other growth factor receptors, we compared our dataset with other available phosphoproteomic datasets. Heibeck and colleagues utilized phosphotyrosine peptide immunoprecipitation and high sensitivity capillary LC-MS/MS to identify 481 basal and EGF-induced tyrosine phosphorylation sites from 285 proteins in a non-transformed human mammary epithelial cell (HMEC) line [31]. From the 98 proteins that showed CSF-1R-induced or CSF-1R-induced* tyrosine phosphorylation (Table 1), there was a 51% (50/98) overlap with EGFR-induced phosphorylated proteins in HMECs (Figure 2B, Table S5). A number of the overlapping proteins (ERK1, ERK2, IRS2, GAB1, SHB, SHC and PI3K p85-alpha) are key signaling intermediates for multiple signaling pathways. The CSF-1R and EGFR pathways also overlap in their regulation of cellular adhesion through the phosphorylation of p120-catenin (p120), cofilin, plakophilin 3, pyk2, talin and integrin β4. Therefore, CSF-1R induces tyrosine phosphorylation of many proteins that are also regulated by EGFR.

Due to the frequent activation/over-expression of ERBB2/HER2 in mammary epithelial tumors, we also examined the overlap between CSF-1R-induced changes in tyrosine phosphorylation and changes induced by this RTK. Phosphoproteomic analysis of tyrosine-phosphorylated proteins in ERBB2-overexpressing breast and ovarian cancer cell lines identified 78 proteins with increased phosphorylation [32], 12 of which overlapped with the CSF-1R- induced and CSF-1R induced* proteins (p120, EPHA2, ACK, integrin β4, HGF-R, KIRREL, caveolin-1, EGFR, GAB1, CDC2, PI3-K p85 alpha, and ERK2). In a similar analysis, Bose and colleagues identified 198 proteins with increased tyrosine phosphorylation in ERBB2-over-expressing NIH-3T3 cells using a quantitative MS approach [33]; only 19 of these overlap with the CSF-1R induced and CSF-1F induced* proteins (annexin A2, β-adaptin, ARP2/3 subunit 3, calmodulin, caveolin, cct-theta, p120, PI3K p85-beta, intersectin 2, LDH-A, PGK1, PKM2, PI3K p85-alpha, PTRF, vimentin, HSP90-β, glutathione-S-transferase, RAN and YES). The lack of extensive overlap with CSF-1R-induced phosphoproteins in both ERBB2 studies could in part reflect cell-type differences (fibroblasts vs. mammary epithelial), species differences (mouse vs. human), and differing receptor activities (ERBB2 vs. CSF-1R). It is also possible that under-sampling contributes to the disparities between the studies due to the nature of MS-based studies.

We identified three proteins with increased tyrosine phosphorylation in all the proteomic datasets. CSF-1R, EGFR and ERBB2 activation all increase the tyrosine phosphorylation of p120, caveolin and PI3-K p85-alpha, suggesting that they are key common substrates of RTK activation. In addition, phosphorylation of ACK, calmodulin, EPHA2, integrin β4, intersectin 2, KIRREL, HGF-R, GAB1, CDC2, ERK2, annexin A2, and PI3-K p85-beta were induced by CSF-1R and identified in at least one of the other three proteomic datasets analyzed for RTK-induced tyrosine phosphorylation indicating that their phosphorylation is not unique to CSF-1R.

From the comparative analysis, we determined that 35 of the CSF-1R induced phosphoproteins did not overlap with those identified in any of the analyzed RTK studies (Table 4). Of these 35 proteins, we were able to link 16 of these proteins to the CSF-1R pathway using Ingenuity IPA, a database of protein-protein interactions curated from the literature, allowing one or two intermediate signaling proteins. However, the remaining 19 of these proteins including ACLY, CNP, UBXD8, GUK 1, H2B1L, H2B2E, LDH-B, KIAA0323, NHP2L1, PDLIM5, PIN4, RPS27, SLC20A1, STEAP1, TAOK1, TJAP1, urdpase1, UQCRC2, and ZDHHC7 could not be linked to the CSF-1R pathway and thus represent novel CSF-1R targets (Table 4). Many of these proteins are not well characterized, and thus could lead to new insights into the biological consequences of CSF-1R activity in epithelial cells and its role in epithelial tumorigenesis.

**Table 4:** Proteins with increased tyrosine phosphorylation unique to CSF-1R.

Protein Name	UniProt #	NCBI Site	Classification
TAOK1	Q7L7X3	Y309	CSF-1R induced
UBXD8	Q96CS3	Y79	CSF-1R induced
UQCRC2	P22695	Y207	CSF-1R induced
SPG20	Q8N0X7	Y46	CSF-1R induced*, Src-dependent
ADK2	P54819	Y199	CSF-1R induced*, Src-independent
D-PROHIBITIN	Q99623	Y121	CSF-1R induced*, Src-independent
GUK1	Q16774	Y52	CSF-1R induced*, Src-independent
KRT81	Q14533	Y282	CSF-1R induced*, Src-independent
LDH-B	P07195	Y239	CSF-1R induced*, Src-independent
PI3K p85-gamma	Q92569	Y373	CSF-1R induced*, Src-independent
STEAP1	Q9UHE8	Y27	CSF-1R induced*, Src-independent
CDK3	Q00526	T14, Y15	CSF-1R induced*, Src-inhibited
NDUFB10	O96000	Y54	CSF-1R induced*, Src-inhibited
PI3-kinase p110 alpha	P42336	Y508	CSF-1R induced*, Src-inhibited
CSF-1R	P07333	Y561	CSF-1R induced, Src-dependent
FLJ20625	Q9NWT0	Y40	CSF-1R induced, Src-dependent
KIAA0323	O15037	Y456	CSF-1R induced, Src-dependent
TJAP1	Q5JTD0	Y352	CSF-1R induced, Src-dependent
ZDHHC7	Q9NXF8	Y130	CSF-1R induced, Src-dependent
INSULIN RECEPTOR	P06213	Y1189	CSF-1R induced, Src-independent
PIN4	Q9Y237	Y122	CSF-1R induced, Src-independent
PKC Delta	Q05655	Y374	CSF-1R induced, Src-independent
RPS27	P42677	Y31	CSF-1R induced, Src-independent
ACLY	P53396	Y1073	CSF-1R induced, Src-inhibited
CNP	P09543	Y110	CSF-1R induced, Src-inhibited
DLG1	Q12959	Y760	CSF-1R induced, Src-inhibited
Enolase 1	P06733	Y188	CSF-1R induced, Src-inhibited
H2B1L	Q99880	Y42	CSF-1R induced, Src-inhibited
H2B2E	Q16778	Y43	CSF-1R induced, Src-inhibited
HGK	O95819	Y1227	CSF-1R induced, Src-inhibited
NHP2L1	P55769	Y31	CSF-1R induced, Src-inhibited
PDLIM5	Q96HC4	Y251	CSF-1R induced, Src-inhibited
SLC20A1	Q8WUM9	Y388	CSF-1R induced, Src-inhibited
UrdPase 1	Q16831	Y35	CSF-1R induced, Src-inhibited
VASP	P50552	Y38	CSF-1R induced, Src-inhibited

### Y561-dependent, putative SRC-dependent, changes in tyrosine phosphorylation

To identify putative SRC targets, we examined whether the detected changes in tyrosine phosphorylation are dependent on Y561 by comparing the phosphotyrosine profile of cells expressing the CA-CSF-1R Y561F mutant, which is unable to recruit SRC, to that of cells expressing the CA-CSF-1R. For the CA-CSF-1R/CA-CSF-1R Y561F comparison, fold change ratios >1.55 were defined as an upregulation in the levels of tyrosine phosphorylation, ratios 0.63–1.55 as unchanged, and ratios <0.63 as a downregulation of tyrosine phosphorylation (See Materials and Methods and Table S3). As Y561 is not exclusively selective for SRC, we cannot distinguish SRC substrates from those of other SFKs, and thus all peptides specifically detected in the CA-CSF-1R Y561F expressing cells are not necessarily exclusively SRC-regulated. It is also possible that other proteins in addition to SRC family kinases can bind to this site; however, we will refer to the Y561-dependent changes as SRC-dependent for simplicity of nomenclature. Tyrosine phosphorylated peptides were found to fall into three different categories based on the Y561 site: “SRC-independent” sites displayed no differences in tyrosine phosphorylation between CA-CSF-1R and CA-CSF-1R Y561F cells; “SRC-dependent” sites demonstrated an increase in tyrosine phosphorylation in CA-CSF-1R cells compared to CA-CSF-1R Y561F cells; and “SRC-inhibited” sites showed higher phosphorylation in the CA-CSF-1R Y561F cells as compared to CA-CSF-1R cells (Table 1, Table S3).

Earlier studies have demonstrated that the CSF-1R Y561F mutation impairs the ligand-dependent auto-phosphorylation of CSF-1R; however, the Y561F mutation did not compromise the auto-phosphorylation of CA-CSF-1R in this analysis as both the activation loop residue, Y809, and Y873 had equal levels of tyrosine phosphorylation between CA-CSF-1R and CA-CSF-1R Y561F [34], [35]. Equal levels of phosphorylation were also detected at the Grb2 binding site, Y699, and Y923, supporting the conclusion that the total kinase activity of CSF-1R was not significantly affected by the Y561F mutation (Table S3). In addition, our previous data indicates that the CA-CSF-1R Y561F is equally competent for signaling as it can induce growth factor independence in MCF-10A cells and promote MCF-10A acini growth similar to CA-CSF-1R [8]. The previously reported defects in autophosphorylation may not be relevant in the context of the active variant of the receptor in which point mutations permit ligand-independent receptor activation and inhibit the receptor downregulation via internalization.

**Figure 3:**
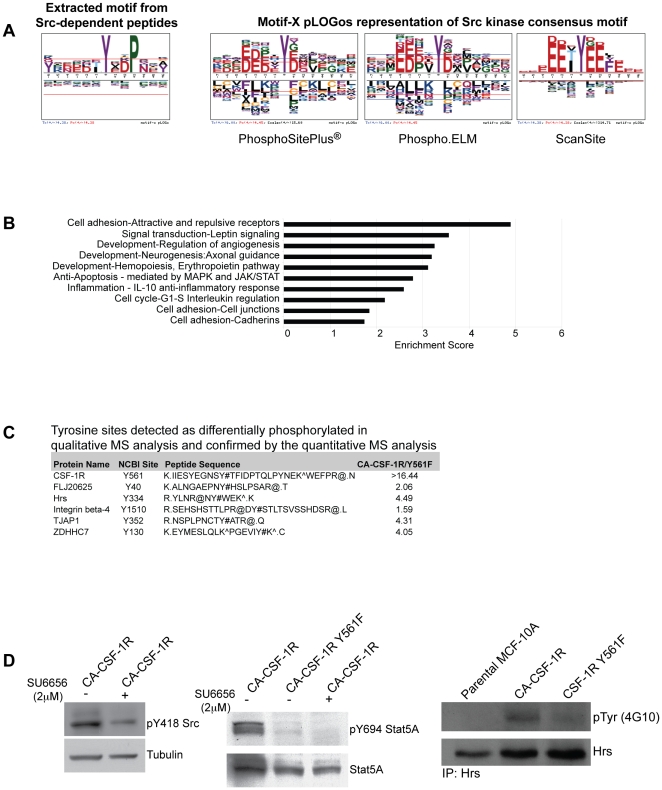
Y561-dependent changes in phosphorylation are SRC-dependent changes in tyrosine phosphorylation. **A**) pLOGos representation of motif extracted from the SRC dependent substrates identified in the quantitative MS analysis. Also shown are motif representations generated from collections of known SRC targets in PhosphoSite® Plus, Phospho.ELM and ScanSite, respectively. **B**) GeneGO® enrichment analysis of the SRC-dependent proteins using the >1.32 inclusive threshold. **C**) Validation of SRC-dependent differential tyrosine phosphorylation detected in a pilot qualitative MS analysis by quantitative MS analysis. Because the pilot experiments did involve the isotopic labeling of cellular proteins, the qualitative MS analysis was designed only to detect the presence of tyrosine phosphorylated peptides isolated by phosphotyrosine immunoprecipitation. **D**) Western blot analysis of SRC, Stat5 and Hrs tyrosine phosphorylation. Lysates from CA-CSF-1R cells treated with either vehicle control or the SRC kinase inhibitor, SU6656, were analyzed for changes in tyrosine phosphorylation using phospho-specific antibodies for SRC Y418 and Stat5 Y694. Hrs immunoprecipitates from Parental, CA-CSF-1R and CA-CSF-1R Y561F MCF-10As were probed with a phosphotyrosine antibody.

Quantitative comparison of the differences in peptide tyrosine phosphorylation between CA-CSF-1R and CA-CSF-1R Y561F-expressing cells identified 22 tyrosine sites on 19 proteins dependent on CA-CSF-1R Y561 that were phosphorylated above threshold (ratio >1.55, see Materials and Methods) (Table 5). Although phosphorylation of SRC at its activation site (Y418) was not detected in cells expressing either form of CSF-1R, CA-CSF-1R was highly phosphorylated at Y561 indicating that this site is competent to recruit and activate SRC. In addition, known SRC targets including p120, ACK, and Hrs demonstrated a Y561-dependent increase in phosphorylation. β-adaptin, intersectin 2, and KIAA1217 were also identified as having SRC-dependent tyrosine phosphorylation sites. Furthermore, this analysis provided supporting evidence of Src regulation of the following sites: Integrin β4 Y1510, FLJ20625 Y40, Hrs Y334, TJAP1 Y352 and ZDHHC7 Y130. These sites were identified as SRC-dependent in CA-CSF-1R expressing MCF-10A cells in a previous non-quantitative LC-MS/MS study (data not shown, Figure 3C).

**Table 5:** SRC-dependent tyrosine phosphorylated peptides identified in the comparison of CA-CSF-1R to CA-CSF-1R Y561F MCF-10A cells.

Protein Name	NCBI Site	Peptide Sequence	Fold change	Function
CSF-1R	Y561	K.IIESYEGNSY#TFIDPTQLPYNEK∧WEFPR@.N	>16.44	Receptor tyrosine kinase
p120catenin	Y904	K.SLDNNY#STPNER.G	5.31	Adhesion
Hrs	Y132	K.VVQDTY#QIMK∧VEGHVFPEFK∧.E	5.06	Adaptor/scaffold
Hrs	Y334	R.YLNR@NY#WEK∧.K	4.49	Adaptor/scaffold
TJAP1	Y352	R.NSPLPNCTY#ATR@.Q	4.31	Adhesion
ZDHHC7	Y130	K.EYMESLQLK∧PGEVIY#K∧.C	4.05	Unknown function
ZDHHC7	Y130	K.EYMESLQLK∧PGEVIY#K∧.C	3.99	Unknown function
ZDHHC7	Y130	K.EYMESLQLKPGEVIY#KCPK.C	3.98	Unknown function
Hrs	Y216	R.VCEPCY#EQLNR.K	3.51	Adaptor/scaffold
Hrs	Y216	R.VCEPCY#EQLNR@.K	3.5	Adaptor/scaffold
Ack	Y518	K.KPTY#DPVSEDQDPLSSDFKR.L	3.45	Protein tyrosine kinase
Hrs	Y132	K.YKVVQDT#YQIMK.V	3.12	Adaptor/scaffold
Hrs	Y132	K.VVQDTY#QIMK∧.V	3.11	Adaptor/scaffold
Intersectin 2	Y552	K.LIY#LVPEK.Q	2.73	Adaptor/scaffold
Hrs	Y132	K.YK∧VVQDTY#QIMK∧.V	2.63	Adaptor/scaffold
ATP1A1	Y260	R.GIVVY#TGDR.T	2.62	Hydrolase
KIAA0323	Y456	R.HIVIDGSNVAMVHGLQHY#FSSR.G	2.22	Unknown function
Beta-adaptin	Y276	K.DSDY#YNMLLK∧.K	2.12	Vesicle protein
Stat 5A	Y694	K.AVDGY#VKPQIK.Q	2.11	Transcription factor
EphA2	Y575	R.QSPEDVY#FSK∧.S	2.11	Receptor tyrosine kinase
EphB4	T587, Y595	K.HGQYLIGHGT#KVYIDPFTY#EDPNEAVR.E	2.09	Receptor tyrosine kinase
FLJ20625	Y40	K.ALNGAEPNY#HSLPSAR@.T	2.06	Unknown function
KIAA0323	Y456	R.HIVIDGSNVAMVHGLQHY#FSSR@.G	1.98	Unknown function
RAN	Y146	K.NLQY#YDISAK.S	1.81	Nuclear receptor co-regulator, G protein
PLEKHA6	Y492	R.SEDIY#ADPAAYVMR@.R	1.79	Lipid binding protein
EphB4	Y774	R.FLEENSSDPTY#TSSLGGKIPIR.W	1.75	Receptor tyrosine kinase
KIAA0323	Y456	R.HIVIDGSNVAM*VHGLQHY#FSSR@.G	1.7	Unknown function
SPG20	Y46	K.GLNTDELGQKEEAKNYY#K.Q	1.66	Unknown function
KIAA1217	Y393	R.NEGFY#ADPYLYHEGR.M	1.6	Unknown function
Integrin β4	Y1510	R.SEHSHSTTLPR@DY#STLTSVSSHDSR@.L	1.59	Adhesion, Receptor

# indicates phosphorylation.

* indicates oxidation.

@ indicates “heavy” arginine.

∧ indicates “heavy” lysine.

53% (171/324) of the peptides detected in the CA-CSF-1R vs. CA-CSF-1R Y561F comparison were classified as SRC-independent (Table S2). Multiple sites on proteins associated with focal adhesions were phosphorylated in a SRC-independent manner, including paxillin Y88, Y118, FAK Y576, p130Cas Y128, Y234, Y267, Y249, Y327, Y387, talin Y70 and integrin β4 Y1207. Since many of these are known targets of SRC, it is likely that SRC molecules activated by integrin receptor engagement, rather than SRC recruitment to CSF-1R, are responsible for these phosphorylation events.

35% (112/324) of peptides detected were classified as SRC-inhibited (Table S2). These peptides contained sites that displayed elevated levels of phosphorylation in CA-CSF-1R Y561F-expressing cells, raising the possibility that SRC/SFKs may negatively regulate these sites. Proteins with phosphorylated tyrosine sites that were higher in the Y561F mutant-expressing cells included caveolin, vimentin, DLG1, and DLG3, among others (Table S3). One possible explanation for this finding is that SRC activates a phosphatase that targets these proteins. Alternatively, it is possible that CSF-1R-induced changes in cellular adhesion could alter the localization of SRC modifying its access to specific substrates and thus leading to distinct patterns of tyrosine phosphorylation dependent on the Y561 site.

### Motif analysis of SRC-regulated peptides

To determine whether the peptides that exhibited putative SRC-dependent phosphorylation sites were enriched for specific sequences flanking the tyrosine phosphoacceptor, we utilized an on-line extraction algorithm, Motif-X (motif-x.med.harvard.edu), developed by Schwartz and Gygi [36], [37]. This algorithm identifies the most frequently occurring residues in positions surrounding the pY and provides statistical information on the significance of their repeated appearance. To identify enriched amino acid sequences in the detected phosphopeptides, peptide sequences were centered around the phosphorylated tyrosine residue, and the significance threshold was set to p<0.0001 with a minimum number of occurrences for a particular sequence set to four because of the relatively small number of peptides analyzed. Because of the limited number of SRC-dependent peptides, we included all peptides with fold change values within one standard deviation of the mean (>1.32). While statistically the detected changes in phosphorylation of peptides with fold change values 1.32–1.55 are less likely to be significant, this set of peptides includes known SRC targets such as p120 and p130Cas, supporting the hypothesis that such peptides may in fact be regulated by CSF-1R-induced SRC activation.

The extracted motif as shown in Figure 3A very closely resembles the previously identified consensus c-SRC kinase substrate motif, D [ED] [EDG] [IVL] Y# [GE] E [FI] F [37]. Recent work by Schwartz and colleagues has led to the development of more informative sequence motif representation using Motif-X pLOGos (probability log-based logos) (Schwartz, Chou and Church, unpublished data). Residues closest to the midline in the pLOGos motif representation have the greatest degree of correlation. Those residues above the midline are overrepresented while those below the midline are underrepresented. The red line indicates a p<0.01. The height of the residue symbol is proportional to the correlation values of the residue at that position, and fixed positions within the sequence are indicated with only one residue.

The motif analysis identified a high selectivity for proline at the +3 position in the SRC-dependent phosphopeptides. Peptides derived from p120, p130Cas, STAT5A, EPHA2, KIAA1217 and ZDHHC7 have a proline at the +3 position. pLOGos representations of SRC consensus motifs derived from annotated substrates in the databases PhosphoSitePlus®, Phospho.ELM and Scansite are shown in Figure 3A [38], [39]. The same preference for a hydrophobic residue at the +3 position appears in each of these motifs. Interestingly, Schwartz and Gygi uncovered this same preference in an analysis of two phosphotyrosine proteomic data sets generated by pervanadate-induced phosphorylation in Jurkat cells and c-SRC Y527F-stimulated phosphorylation in NIH-3T3 cells [23], [36]. The SRC consensus representations also clearly indicate a preference for acidic residues at positions −4, −3, −2, and +1; the motif extracted from the CSF-1R-induced SRC substrates has the same profile. Thus, while the nature of this study does not allow us to distinguish between direct and indirect SRC substrates, this motif analysis of the Y561-dependent phosphorylation sites suggest that many are in fact direct SRC substrates since the most prominent sequence motif closely matches known c-SRC consensus motifs [36], [37]. In addition, the motif analysis validated the inclusion of phosphopeptides with fold change values 1.32–1.55 and provided evidence that these sites may be SRC regulated since many closely match the SRC consensus motif.

### Enrichment Analysis of the SRC-dependent peptides

To derive information on the cellular functions of the SRC-dependent substrates, we performed an enrichment analysis. This analysis included all SRC-dependent phosphorylated proteins with a fold change >1.32 (Table S2). Three of the cellular processes found to be overrepresented in this set of substrates were related to some aspect of cell adhesion including the regulation of cell junctions and cadherins (Figure 3B). The enrichment identified p120, plakophilin 4, erbin, EPHA2, EPHB4, and PIK3R3 as proteins associated with the regulation of cell junctions and cadherins; these proteins may be candidate mediators of the CSF-1R-induced SRC-dependent adhesion disruption.

### Validation of differential tyrosine phosphorylation

While it wasn't feasible to validate a large number of tyrosine phosphorylation sites identified in this screen, six of the proteins identified in the SILAC analysis overlap with SRC-dependent substrates identified in a pilot, non-quantitative MS analysis (Figure 3C and data not shown). We also validated the SRC-induced tyrosine phosphorylation of two substrates, Hrs and STAT5A (Figure 3D). CA-CSF-1R-expressing MCF-10As were treated with the SRC kinase inhibitor, SU6656, overnight prior to analysis. Treatment with the inhibitor decreased phosphorylation of the SRC active site (Y418) (Figure 3D), validating the efficacy of the inhibitor. Tyrosine phosphorylation of STAT5A Y694 was reduced in the SU6656-treated CA-CSF-1R cells, and the CSF-1R Y561F did not induce phosphorylation of the site. In addition, we observed that tyrosine phosphorylation of Hrs was not induced by the CSF-1R Y561F. While we were unable to define which sites on Hrs were phosphorylated, this evidence validates that phosphorylation of this protein is SRC-dependent.

### Analysis of tyrosine phosphorylation in human cancer phosphoproteomic datasets

To address the relevance of the identified SRC substrates to tumorigenesis, we conducted comparative bioinformatics analysis on phosphoproteomic datasets generated from human tumor samples. Because we profiled CSF-1R-induced and SRC-dependent changes in tyrosine phosphorylation in a mammary epithelial cell line, we first analyzed a small breast cancer dataset, derived from only eight tumors, available publicly from CST's PhosphoSitePlus® [38]. Many SRC-dependent sites identified in our study were tyrosine phosphorylated in these human breast tumors (Table 6). The small size of the dataset and the detection of activated SRC in all eight tumors precluded a rigorous correlation analysis between SRC activation and substrate phosphorylation. However, several putative SRC substrates, including HIPK3, STAT5A, p120, FRK and Hrs, were frequently phosphorylated in multiple breast tumors.

**Table 6:** Comparative analysis of tyrosine phosphorylation detected in human breast tumors.

Protein Name	NCBI site	Fold change (CA-CSF1R/Y561F)	# of tumors	Tumors* with phosphorylation
Src	Y418	-	8	1, 2, 3, 4, 5, 6, 7, 8
HIPK3	359	1.43	8	1, 2, 3, 4, 5, 6, 7, 8
STAT5A	694	2.11	7	2, 3, 4, 5, 6, 7, 8
p120catenin	904	5.31	6	1, 2, 4, 5, 6, 8
Hrs	216	3.51	5	1, 3, 4, 5, 8
Frk	46	1.41	5	1, 3, 6, 7, 8
Ack	518	3.45	4	1, 4, 5, 6
Hrs	132	5.06	2	2, 4
ATP1A1	260	2.62	1	2
p120catenin	228	1.53	1	2

*Tumor# PhosphoSite Plus® ID.

1  BC0001.

2  BC0002.

3  BC0003.

4  BC0004.

5  BC0005.

6  BC0007.

7  BC0008.

8  csBC0001.

Given the limited amount of phosphotyrosine proteome data available from breast tumor dataset, we also analyzed a larger set of phosphoproteomic data derived from human non-small cell lung cancer. Rikova and colleagues analyzed the phosphorylation of proteins in 150 human NSCLC lung tumors using PhosphoScan® analysis [40]. Activating mutations of *EGFR* are frequently found in NSCLC tumors, and EGFR activation can similarly induce the upregulation of endogenous c-SRC. We performed correlation studies to determine the substrates whose phosphorylation significantly correlated with SRC activity based on detection of the phosphorylation of Y418 SRC autophosphorylation site.

The comparison revealed that many SRC-dependent proteins were also tyrosine phosphorylated in human lung tumors. Five proteins (p120, p130Cas, FRK, Hrs and KIAA0323) showed a significant correlation (p-value<0.05) between SRC activation and tyrosine phosphorylation (Table 7). Three of these five proteins (p120, Hrs, and FRK) were also commonly tyrosine phosphorylated in the human breast cancer dataset. This finding of significant co-regulation of SRC activity and substrate phosphorylation indicate that these may be critical SRC substrates in the context of a human epithelial tumor. Together, our analyses suggest that a subset of the SRC-dependent substrates we identified downstream of CSF-1R induced SRC activation are relevant in human epithelial tumorigenesis. Substrates such as p120, Hrs, Ack, p130Cas and FRK may be key mediators of SRC signaling in human tumors and contribute directly to SRC-mediated events during tumor progression.

**Table 7:** Comparative analysis of tyrosine phosphorylation detected in human lung tumors.

Protein Name	NCBI Site	Fold change (CA-CSF-1R/Y561F)	p-Value
p130Cas	Y234	1.37	0.001993
p120catenin	Y228	1.53	0.004847
p120catenin	Y904	5.31	0.013537
Frk	Y46	1.41	0.017747
Hrs	Y216	3.5	0.021389
KIAA0323	Y502	2.22	0.037628

## Discussion

The quantitative MS analysis performed in this study identified proteins phosphorylated downstream of CSF-1R activation in mammary epithelial cells and defined a specific set of these proteins as candidate targets of SRC family kinases. While earlier studies have identified CSF-1R and SRC targets phosphorylated in other cell types, some of which were detected in this analysis, previously unrecognized targets of these kinases were also revealed in this study. Comparative analysis with other RTK tyrosine phosphoproteomic datasets revealed multiple overlapping pTyr substrates in CSF-1R-, EGFR- and ERBB2-expressing cells and highlighted the shared regulation of caveolin, p120 and PI3-K by these RTK pathways. Many of the putative SRC substrates including p120, Hrs and STAT5A are likely direct SRC targets based on the degree of overlap between the peptides detected and the known SRC consensus motif, as well as the observed downregulation of tyrosine phosphorylation by pharmacological inhibition of SRC kinase activity. In addition, the phosphorylation of several SRC substrates significantly correlated with SRC activation in human lung tumors. Substrates that correlated with SRC activation, such as p120, Hrs, p130Cas, FRK and KIAA0323, may be critical mediators of SRC activity in human tumors.

### CSF-1R signaling

The CSF-1 pathway is required for macrophage differentiation and survival, yet previous work suggests the CSF-1 pathway may also drive tumor cell progression. Our data delineate the changes in tyrosine phosphorylation induced by CSF-1R signaling in epithelial cells. Unlike substrates of EGFR and ERBB2, CSF-1R targets in epithelial cell have not been well characterized. Like other growth factor receptor tyrosine kinases, CSF-1R induces pleiotropic phenotypic alterations in epithelial cells. CSF-1R activation in MCF-10A cells induces cell-cell adhesion disruption, loss of contact inhibition, growth in soft agar, and enhanced cell motility [8], [41]. In addition, recent studies showed that invasion by a human breast tumor cell line is dependent on paracrine signaling with host macrophages as well as autocrine signaling involving CSF-1R expressed on tumor cells themselves [14]. The candidate substrates identified in our analysis (e.g. p120, integrin β4, plakophilin 3, STAT3 and vimentin) will inform future studies addressing the mechanisms involved in CSF-1R-induced tumor progression.

Using comparative bioinformatic analysis, we found that CSF-1R modified a set of proteins which did not overlap with those phosphorylated downstream of other RTKs e.g., EGFR and ERBB2. From this set of 35 proteins, we identified 19 proteins, which had not been previously linked to CSF-1 signaling based on studies published to date; these proteins may represent substrates unique to CSF-1R signaling. These proteins regulate a number of different cellular processes including trafficking, cell cycle progression and cell polarity and exhibit such diverse functions as kinases, adaptors and structural proteins. Many of these proteins are not well characterized; thus studies of these putative novel targets may reveal new mechanistic insights into epithelial cell biology and tumorigenesis. For example, two of these proteins, ACLY and UQCRC2, play a role in the regulation of cellular metabolism. ACLY, ATP citrate lyase, is an enzyme involved in the conversion of glucose to cytosolic acetyl CoA, which is a part of the glucose-dependent lipogenesis pathway [42], [43]. UQCRC2, ubiquinol-cytochrome C reductase complex III protein 2, is a protein involved in oxidative phosphorylation and is a subunit of the mitochondrial cytochrome bc1 complex. Cancer cells undergo changes in metabolism in order to shift to the metabolic demands of proliferative cells [44], [45]. Interestingly, ACLY can be phosphorylated and activated by Akt [46]. Upregulation of ACLY activity in tumor cells expressing activated Akt could suppress fatty acid oxidation (through increased acetyl-CoA and malonyl-CoA production), thus supporting the significant requirement for lipid synthesis in proliferative cell states. Serine phosphorylation of ACLY in non-small cell lung cancer cell lines has been shown to upregulate ACLY activity and siRNA-mediated KD of the protein induces growth arrest [43]. ACLY expression is increased in metastatic breast cancer lines in comparison to non-tumorigenic and non-metastatic cell lines [42]. These data could suggest a role for ACLY in breast metastasis and its phosphorylation in the regulation of protein function. In our study, CSF-1R activation induced a 26-fold increase in tyrosine phosphorylation of ACLY at Y1073. Our data may reveal new insights into the regulation of ACLY by tyrosine phosphorylation. Similarly, UQCRC2 levels are altered in cancer cells, showing a decrease in mitochondria from breast cancer cells as compared to normal breast cells [47]. Since we detected a 10-fold increase in CSF-1R-dependent phosphorylation of UQCRC2, this raises that possibility that this modification may downregulate it's activity, thus causing a similar loss of activity as observed in tumor cells with lower levels of UQCRC2 expression. The changes in tyrosine phosphorylation on proteins involved in metabolism highlight candidate effectors that may contribute to altered metabolism in CSF1-1R-driven phenotypes.

### CSF-1R-induced, SRC substrates

As the first characterized proto-oncogene, SRC has been extensively studied in human tumors; however, SRC itself is not frequently mutated, and on its own is not strongly transforming. The increased activation of SRC detected in tumor cells may therefore result from upstream activated RTKs [10]. CSF-1R-induced SRC activation may mimic the upregulation of SRC activity by RTKs in human tumors. Therefore, CSF-1R-induced SRC-dependent substrates are likely more relevant to tumorigenesis than substrates of overexpressed c-SRC or activated mutants of the protein. Surprisingly, comparison of SRC substrates identified in our study to SRC targets identified in other MS-based studies revealed relatively small overlap (data not shown) [22], [23], [24], [25], [26]. This may be due to the use of overexpressed or constitutively active SRC in these studies, which may lead to phosphorylation of a broader set of substrates distinct from those targeted by RTK-induced SRC activity. SRC can be activated by multiple classes of membrane receptors, such as G protein-coupled receptors, cell adhesion molecules, and integrins. Overexpression of wild-type or activated SRC mutants may induce phosphorylation of this broader subset of targets distinct from the substrates of endogenous c-SRC activated by an upstream growth factor receptor.

SRC activity and substrate phosphorylation is tightly controlled in vivo by intramolecular interactions and protein localization, and such regulatory mechanisms are disrupted in SRC mutant variants [5]. In our study, endogenous wild-type SRC was activated by an upstream RTK, more closely reflecting the effects of activation of endogenous SRC in tumor cells. Thus, our experimental design facilitated the selective identification of targets most likely regulated by RTK-activated SRC. Furthermore, we identified five proteins whose phosphorylation was significantly associated with SRC activation in human lung tumors. Of these five proteins, p120, Hrs and FRK may be particularly relevant to SRC-driven tumor progression. The SRC-related tyrosine kinase, FRK, was recently demonstrated to act as tumor suppressor by attenuating AKT/PI3Kinase signaling through its action on the phosphatidylinositol-3,4,5-trisphosphate 3-phosphatase, PTEN [48]. FRK-mediated phosphorylation of PTEN blocks its interaction with an E3 ubiquitin ligase and stabilizes PTEN protein. The siRNA-mediated knockdown of FRK in MCF-10A cells resulted in depletion of PTEN, and its loss was sufficient to cause transformation [49]. As such, our finding of a significant correlation between SRC activity and FRK tyrosine phosphorylation in human lung tumors raises the possibility that SRC-mediated phosphorylation of FRK may negatively regulate FRK functions.

### CSF-1R-SRC-induced adhesion disruption – regulators of E-cadherin surface stability

Our study aimed to define the CSF-1R induced, SRC-regulated proteins in part to identify candidate SRC substrates that may regulate cell adhesion in MCF-10A cells [8]. RTK-induced SRC activation may also be responsible for the regulation of specific human tumor phenotypes such as cell invasion and motility, which can increase the metastatic potential of a tumor, as SRC targets substrates involved in the processes of cellular adhesion and migration [50]. Elevated SRC activity is associated with invasiveness in colon tumors, suggesting that SRC induces cellular changes that promote tumor progression [11]. A key component of invasive potential is the downregulation of cell-cell adhesion and the adhesive strength of adherens junctions (AJs), which can be accomplished by reducing surface expression of E-cadherin [51]. The oncogenic mutant v-SRC can induce the downregulation of E-cadherin from the plasma membrane through tyrosine phosphorylation and ubiquitination, leading to E-cadherin degradation in lysosomes [52], [53]. However, our previous work revealed that E-cadherin levels do not decrease in the presence of SRC activation by CSF-1R in MCF-10As, but rather E-cadherin is redistributed to intracellular vesicles. Thus, we hypothesized that E-cadherin is sequestered in the cytoplasm following CSF-1R induction of SRC activity [8].

p120 likewise regulates the stability of E-cadherin and is a well-characterized SRC target [54]. In our study, CSF-1R induced the tyrosine phosphorylation of p120 Y904 and Y228 in a SRC-dependent manner (Table 5). Given the evidence that p120 is a key regulator of cadherin stability and localization, these phosphorylation events may be critical to the regulation of p120 function and activity. p120 has multiple tyrosine residues that can be modified by SRC; yet the functional consequences of phosphorylation on p120 function are not well understood [55], [56]. We observed a difference in the relative increase of SRC-dependent phosphorylation between Y904 (5.31 fold increase) and Y228 (1.53 fold). Little is known about the significance of phosphorylated Y904, however, it has been detected in multiple whole-cell MS-based analyses (PhosphoSite, http://www.phosphosite.org/home). siRNA-mediated depletion of p120 dramatically disrupts cell-cell adhesion and reduces the levels of E-cadherin and associated proteins at AJs [57]. Similarly, CSF-1R activation of SRC leads to impaired cell-cell adhesion and re-localization of E-cadherin. As SRC phosphorylation of p120 correlates with disruption of MCF-10A adhesion, this modification may impair its function. SRC may further regulate p120 by modulation of the stoichiometry of p120 phosphorylation at specific sites.

Plakophilin 4, a p120 family member that exhibits cell type-dependent localization to AJs and desmosomes [58], is also tyrosine phosphorylated downstream of CSF-1R activation in a SRC-dependent manner (Table S2). Overexpression of plakophilin 4 increases surface expression of classical cadherins, and plakophilin 4 functions similarly to p120 to stabilize cell-cell junctions by regulating E-cadherin [54]. Intriguingly, plakophilin 4 interacts with erbin [58], which also demonstrated increased phosphorylation at Y1104 in our study. Their coordinate phosphorylation may be involved in SRC regulation of cell-cell adhesion.

SRC may also influence the dynamics of cellular endocytosis indirectly leading to the downregulation of E-cadherin. Hrs, β-adaptin, and intersectin 2 demonstrated a SRC-dependent increase in tyrosine phosphorylation. These proteins have been previously shown to regulate distinct steps in endocytic trafficking [59], [60], [61]. Hepatocyte growth factor-regulated tyrosine kinase substrate (Hrs) is an important regulator of protein sorting at the endosome. Residing at the cytoplasmic face of early endosomes and multivesicular bodies, Hrs interacts with multiple proteins such as clathrin and EPS15 in addition to mono- and poly-ubiquitinated proteins via its ubiquitin-interacting motif (UIM) [61], [62], [63]. Palacios and colleagues further demonstrated that E-cadherin sorting to the lysosome requires an intact Hrs UIM, and E-cadherin was found to associate with Hrs in an ubiquitin-dependent manner [52]. As with p120, our data suggest that SRC regulation of Hrs may be complex, as multiple tyrosine residues within Hrs were modified and to varying levels.

### SRC activation and substrates in human tumors

Analysis of the overlap between substrates phosphorylated in our study and in human tumors defined a group of proteins that may be most relevant to Src activity in human cancers: HIPK3, STAT5A, p120, Hrs and FRK in human breast tumors, and p130Cas, p120, FRK, Hrs and KIAA0323 in human lung tumors. Each of these proteins has been previously implicated in cancer onset or progression, and our data suggest that their tyrosine phosphorylation may play a role in oncogenic changes. SRC activity in human tumors may further coordinate the phosphorylation of multiple substrates to mediate downstream phenotypes. For example, the coordinate phosphorylation of p120 and Hrs could be required to significantly impact E-cadherin surface stability and disrupt cell-cell adhesion. The identification of a critical set of SRC substrates may lead to the definition of a SRC signature that could serve as a molecular flag that SRC is active in a given tumor. Thus, while monitoring the phosphorylation of one or two SRC targets may not predict response to a clinical SRC inhibitor like dasatinib, phosphorylation of a collection of proteins may be a more useful predictor of efficacy.

## Materials and Methods

### Chemicals and Antibodies

Stable isotope containing amino acids ^13^C_6_-^15^N_2_-L-lysine and ^13^C_6_-^15^N_4_-L-arginine were purchased from Cambridge Isotope Labs (Andover, MA). Antibodies to SRC phospho-Y418, STAT5A phospho-Y694, and STAT5A, were purchased from Cell Signaling Technologies (Danvers, MA). The tubulin antibody was obtained from Abcam Inc. (Cambridge, MA), and the Hrs antibody was procured from Alexis Biochemicals/Enzo Life Sciences (Farmingdale, NY). SRC inhibitor, SU6656, (Calbiochem) was used at 2µM.

### Cell culture and stable isotope labeling with amino acids in culture (SILAC)

CA-CSF-1R and CA-CSF-1R Y561F MCF-10A cells were generated using pMSCV human CSF-1R L301S/Y969F provided by O. Witte (University of California, Los Angeles, CA). The Y561F mutant receptor was generated previously by Carolyn Wrobel [8]. MCF10A cells were grown in a DMEM/F12 based medium as described previously [64] supplemented with dialyzed horse serum and either “light” or “heavy” arginine and lysisne. Cells were labeled over the course of five passages in the labeling medium prior to analysis. Prior to lysis, cells were cultured overnight in MCF-10A growth medium supplemented with reduced (2%) serum and deficient for EGF. Cells were lysed (9M urea, 20mM HEPES pH 8.0, 2.5mM sodium pyrophosphoate, 1mM beta glycerophosphate, and 1mM sodium vanadate). The lysates were sonicated and cleared by centrifugation in preparation for protease digestion, phophotyrosine immunoprecipitation and tandem liquid chromatography mass spectrometry.

### Phosphopeptide immunoprecipitation

Phosphopeptides were prepared using PhosphoScan® Kit (Cell Signaling Technology).

### Analysis by LC-MS/MS Mass Spectrometry

Peptides in the immunoprecipitation eluate (40 µl) were concentrated and separated from eluted antibody using Stop and Go extraction tips (StageTips) [65]. Peptides were eluted from the microcolumns with 1 µl of 60% MeCN, 0.1% TFA into 7.6 µl of 0.4% acetic acid/0.005% heptafluorobutyric acid (HFBA). The sample was loaded onto a 10 cm×75 µm PicoFrit capillary column (New Objective) packed with Magic C18 AQ reversed-phase resin (Michrom Bioresources) using a Famos autosampler with an inert sample injection valve (Dionex). The column was developed with a 45-min linear gradient of acetonitrile in 0.4% acetic acid, 0.005% HFBA delivered at 280 nl/min (Ultimate, Dionex). Tandem mass spectra were collected in a data-dependent manner with an LTQ ion trap mass spectrometer (ThermoFinnigan), using a top-ten method, a dynamic exclusion repeat count of 1, and a repeat duration of 10 sec. TurboSequest (ThermoFinnigan) searches were done against the NCBI human database released on August 24, 2004 (containing 27,175 proteins) allowing for tyrosine phosphorylation (Y+80) and oxidized methionine (M+16) as dynamic modifications. Sequence assignments were accepted based on the score-filtering criteria we described previously (Rush et al., 2005), except assignments that had the score characteristics of false-positive assignments, determined by searching against a database of reversed protein sequences [66] were not accepted.

To guide our assignment of the threshold for significant fold changes phosphorylation, we first graphed the distribution of the non-phosphorylated peptides present in the immunoprecipitated samples and determined the mean of the quantitative fold change values. We assumed data normality based on these distributions; therefore, fold change values greater than two deviations from the mean are considered 95% likely to represent significant differences in tyrosine phosphorylation. Each comparison had a different mean value for the non-phosphopeptide distribution; however, the same statistical threshold was applied to both comparisons. For the CA-CSF-1R/parental MCF-10A comparison, fold change ratios >1.94 were defined as an upregulation in the levels of tyrosine phosphorylation, ratios 0.64–1.94 as unchanged, and ratios <0.64 as a downregulation of tyrosine phosphorylation. For the CA-CSF-1R/CA-CSF-1R Y561F comparison, fold change ratios >1.55 were defined as an upregulation in the levels of tyrosine phosphorylation, ratios 0.63–1.55 as unchanged, and ratios <0.63 as a downregulation of tyrosine phosphorylation. All of the detected phosphopeptides were classified for CSF-1R and SRC regulation on the basis of the calculated relative fold differences (Table S3).

### Immunoprecipitation and Western blot analysis

Cells were lysed in RIPA lysis buffer supplemented with protease inhibitors and phosphatase inhibitors. Lysates were run on an SDS 10% polyacrylamide gel and transferred onto PVDF membranes (Millipore, Bedford, MA), and immunoblots were visualized with an enhanced chemiluminescence kit (Perkin Elmer, Boston, MA).

### Phosphorylation Motif Analysis Using Motif-X

The Motif-X algorithm [36] was used to extract significantly enriched phosphorylation motifs from the phosphopeptide data set. The identified peptides were centered at the phosphorylated amino acid and aligned by the algorithm using the human MS proteome. The probability threshold was set to P<10^−4^, and the occurrence threshold was set to a minimum of four peptides.

### Biological process enrichment analysis

Proteins identified from the PhosphoScan® analysis were assessed for enrichment of Gene Ontology and GeneGO® categories using GeneGO® software (http://www.genego.com). The enrichment analysis computes the probability that a set of genes that are annotated within a specific category would occur by chance and the results are given in the form of p-values. The enrichment score provided on bar graphs is computed as −log(p-value).

### Comparative Bioinformatics Analysis

Phosphorylated proteins from the CSF-1R/SRC PhosphoScan® analysis were compared to published mass spectrometry datasets using BioConductor/R (http://www.bioconductor.org/). SwissProt identifiers from the CSF-1R study were mapped to human and mouse Entrez Gene Identifiers and merged to Entrez Gene Identifiers mapped identifiers provided in published studies. Networks showing the degree of overlap were generated in Cytoscape using the Cerebral Plug-in. Overlap with published tumor data sets was performed with a custom Perl script that identifies overlapping peptides on the basis of peptide sequence. Correlation p-values were calculated using the right-sided Fisher's exact test. To determine whether the phosphorylated proteins from the CSF-1R/SRC PhosphoScan® analysis have been previously linked to CSF-1R signaling, a comprehensive CSF-1R protein interaction network was generated using the Grow Tool in Ingenuity Pathways Analysis (Ingenuity® Systems, www.ingenuity.com). The genes from this network were cross-referenced to the proteins from the PhosphoScan® analysis.

## Supporting Information

Table S1 Tyrosine phosphorylated peptides identified in the comparison of CA-CSF-1R to parental MCF-10A cells.
(0.12 MB XLS)

Table S2 Tyrosine phosphorylated peptides identified in the comparison of CA-CSF-1R to CA-CSF-1R Y561F MCF-10A cells.
(0.12 MB XLS)

Table S3 Classification of CSF-1R- and Src-regulated changes in tyrosine phosphorylation in MCF-10A cells.
(0.07 MB XLS)

Table S4 GeneGO® enrichment in the CSF-1R induced and CSF-1R induced* set of proteins for biological processes and diseases.
(0.04 MB XLS)

Table S5 Proteins with increased tyrosine phosphorylation downstream of CSF-1R and EGF-R activation.
(0.03 MB XLS)
